# Current methods and attitudes of women towards contraception in Europe and America

**DOI:** 10.1186/1742-4755-10-7

**Published:** 2013-02-05

**Authors:** Sarah Johnson, Christine Pion, Victoria Jennings

**Affiliations:** 1 Clinical and Medical Affairs Manager, SPD Development Company Limited, Priory Business Park, Bedford MK44 3UP, United Kingdom; 2 SPD Swiss Precision Diagnostics GmbH, Geneva, Switzerland; 3 Institute for Reproductive Health, Georgetown University, Washington, USA

**Keywords:** Contraception, Contraceptive pill, Condoms, Natural family planning, Persona®

## Abstract

**Background:**

The choice of available contraceptive methods has increased in recent years; however, recent data on women’s awareness of methods and reasons for their method choice, or reasons for changing methods, is limited. The aim of this study was to examine the use and awareness of contraceptive methods in the USA, UK, Germany, Italy and Spain.

**Methods:**

Quantitative survey of heterosexual women aged 25–44 years (n=2544), with no known infertility. Questions related to knowledge and use of contraceptive methods, reasons for choice and for changing methods, and sources of advice.

**Results:**

There was generally good awareness of most forms of contraception in all five countries. Awareness and current usage was greatest for the contraceptive pill (awareness >98%, usage varied from 35% [Spain] to 63% [Germany]); and male condom (awareness >95%, usage varied from 20% [Germany] to 47% [Spain]); awareness of other methods varied between countries. Doctors have the greatest influence on women’s choice of contraceptive method (>50% for all countries), and are most likely to suggest the contraceptive pill or male condom.

Women’s contraceptive needs change; 4–36% of contraceptive pill users were likely to change their method within 12 months. For previous contraceptive pill users (n=377), most common reason for change was concern about side effects (from 26% [Italy] to 10% [UK]); however, awareness of many non-hormonal contraceptive methods was low.

**Conclusions:**

Women aged 25–44 are aware of a wide variety of contraceptive methods, but knowledge and usage of the contraceptive pill and condoms predominates. Changing contraception method is frequent, occurring for a variety of reasons, including change in life circumstances and, for pill users, concerns about side effects.

## Background

The choice of available contraceptive methods has increased in recent years, yet the contraceptive pill, first introduced in the 1960s, remains the method of choice for many women in Europe and the United States of America (USA) [1,2]. Furthermore, despite this increase in choice of contraceptive methods the incidence of unwanted pregnancies remains high in many countries. For example, 49% of all pregnancies were unintended in the USA in 2001 [3]. Overall this rate is unchanged since 1994, but the rate of unintended teenage pregnancies has declined, while the rate has increased among adults aged 25–34 years. This suggests that many women have not identified an ideal method of contraception which fits their lifestyle and meets their personal requirements. Women could potentially benefit from increased information and advice on contraception to ensure their chosen method best suits their individual needs. Recent data on women’s awareness of methods of contraception and reasons for their method choice, or reasons for changing methods, is, however, limited; therefore it is not clear what information and advice may be needed.

This study sought to obtain information on the use and awareness of different forms of contraception among women in the United Kingdom (UK), Germany, Italy, Spain and the USA. This was a questionnaire-based study, which explored women’s knowledge and use of various forms of contraception, why women change their contraceptive method and the reasons for their choice. The study also aimed to determine where women seek advice and whom they consult in making their decisions.

## Methods

Women aged 25–44 years, who were not known to suffer from infertility, participated in an online survey in the UK, Germany, Spain, and the USA. In Italy interviews were carried out at volunteers’ homes, due to less widespread internet usage at the time of the survey (2010). The survey was conducted and analysed by IPSOS based on criteria supplied by the authors.

Women were recruited from an online panel, which due to the very high number of individuals registered, provides a representative sample from each country. For example the US panel contains over 1 million volunteers willing to participate in surveys. Demographic data is collected when volunteers join the panel; therefore volunteers can be selected from the panel based on the inclusion/exclusion criteria stipulated for a particular survey. For this survey, the inclusion criteria were aged 25–45 and able to bear children, and exclusion criteria were professions associated with market research and medical professionals. Volunteers received reimbursement for their participation in the form of ‘survey points’, which can be used to select a gift.

A minimum of 500 women were recruited from each country, providing a sample size sufficient to examine use and awareness of contraceptive methods [4]. Questionnaires were designed to assess current levels of usage and awareness of different forms of contraception, reasons for choosing or changing methods and sources of information and advice. The majority of questions were closed where volunteers had to select from a list of possible answers, but some open-ended questions were also included where the volunteer was asked to provide her views on, or experience of, a method. For example when considering awareness of different forms of contraception, the question was phrased in three different ways, with two open questions followed by a closed question: ‘When thinking about methods of birth control, what ONE method comes first to your mind?’ and ‘And what are ALL the other methods of birth control you have ever heard of?’ followed by a closed question ‘From the list of birth control methods below, please select the ones you have EVER HEARD OF or READ about’. The first two questions provide an estimation of the unprompted awareness among women of forms of contraception, whereas the third question, selecting from a list, provides the prompted awareness level. All questionnaires were translated into the appropriate native language.

Data from online interviews was automatically entered into the Quantum study database. For the face to face interviews, data was entered directly onto a laptop by the interviewer then transferred to the database. Volunteers could only complete the questionnaire once, and as volunteers were contacted directly, duplication was not possible. To complete the questionnaire, all questions had to be answered in order. The number and percentage of responses per category were calculated.

## Results

Questionnaire results were obtained from approximately 500 women in each of the five participating countries; UK, Germany, Spain, Italy and the USA. The average age of women was 35 years and approximately 70% were either married or living with a partner (Table 1).

**Table 1:** Details of study population

	**UK (n=510)**	**Germany (n=514)**	**Spain (n=510)**	**Italy (n=503)**	**USA (n=507)**
Average age, years (SD)	35.0 (5.6)	35.2 (5.7)	34.7 (5.5)	34.9 (6.1)	34.6 (5.7)
Married/living together	67%	73%	73%	70%	76%
Average number of children	1.38	0.98	0.97	0.95	1.48

*SD*, standard deviation.

### Current contraceptive use

**Figure 1:**
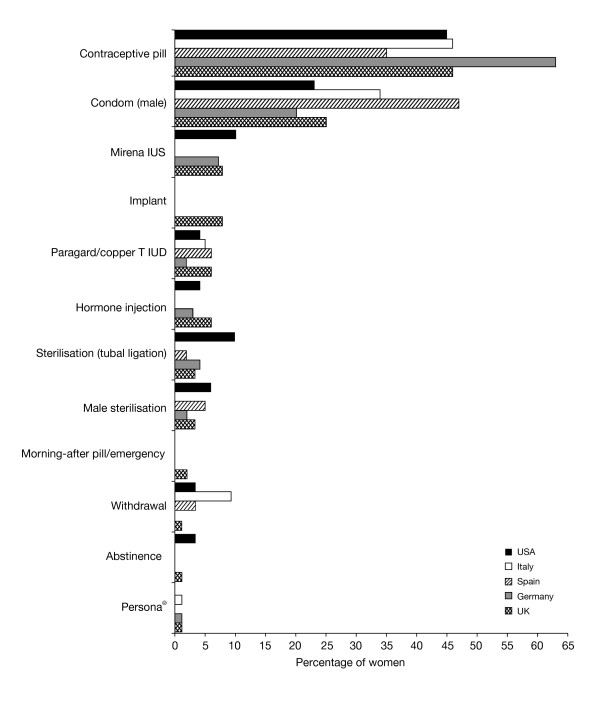
**Country-specific current methods of contraception used by women (percentage of women currently using contraception).** UK, n=313; Germany, n=321; Spain, n=308; Italy, n=342; USA, n=262 (note Persona® is not available in the USA; only methods where there was ≥1% usage are shown).

The most common contraceptive methods currently used by respondents in all countries were the contraceptive pill and male condom. Contraceptive pill use varied between 35% in Spain and 63% in Germany and use of condoms varied between 20% in Germany and 47% in Spain (Figure 1). The contraceptive pill was the most commonly used method in all countries except Spain, where condoms were more commonly the method of choice. The most commonly stated reasons for choosing the contraceptive pill and condoms are shown in Table 2. Current use of all other contraceptive methods was low (reported by ≤10% of responders). A variety of natural family planning (NFP) methods were reported separately, with only Persona® (a luteinising hormone and oestrogen detecting home-use monitor, for identification of days when abstinence is required to avoid pregnancy; Swiss Precision Diagnostics) having ≥1% usage in any country as a single NFP method. Overall, approximately 80% of women were satisfied with their current method of contraception and 27% (in Italy) to 54% (in Germany) stated that they were very satisfied (Table 3). The most common reasons given for dissatisfaction with the contraceptive pill were not wanting to use hormones, weight gain and mood swings. Reasons for dissatisfaction with using condoms were, wanting more security, allergy to latex or inconvenience.

**Table 2:** Stated reasons for choosing the contraceptive pill and condoms as methods of contraception

	**UK**	**Germany**	**Spain**	**Italy**	**USA**
**The contraceptive pill,%**	(n=144)^a^	(n=202)^a^	(n=107)^a^	(n=158)^a^	(n=83) ^a^
Reliable in preventing pregnancy	90	90	85	87	83
Easy to use	75	69	64	53	81
Comfortable for me	56	64	64	54	60
Recommended by doctor	53	56	62	61	64
Inexpensive	44	9	24	6	43
No preparation required	44	42	50	23	53
**Male Condoms**	(n=77)^a^	(n=65)^a^	(n=144)^a^	(n=115)^a^	(n=61)^a^
Reliable in preventing pregnancy	70	58	76	56	70
Minimal or no side effects	58	55	67	53	75
Easily available	57	65	64	40	80
Protects against sexually transmitted diseases	53	60	64	40	54
Causes fewer health concerns than other methods	49	57	60	42	67
Easy to use	48	58	67	50	75

^a^Number of women responding to the question.

**Table 3:** Women’s experiences with their current and previous methods of contraception

	**UK**	**Germany**	**Spain**	**Italy**	**USA**
**Satisfaction with current method,%**	(n=507)^a^	(n=514)^a^	(n=510)^a^	(n=503)^a^	(n=507)^a^
Very satisfied	53	54	41	27	52
Satisfied	31	33	41	57	26
Neither satisfied or dissatisfied	9	8	7	9	12
Experienced side effects*,%					
Current method (n)^a^	18 (313)	9 (321)	16 (308)	5 (342)	20 (262)
Previous method (n)^a^	51 (491)	29 (501)	45 (468)	26 (479)	52 (465)
With contraceptive pill (n)^b^	78 (258)	79 (148)	87 (217)	87 (131)	81 (244)
With hormone injections (n)^b^	24 (258)	9 (148)	1 (217)	0 (131)	18 (244)
With implants (n)^b^	7 (258)	3 (148)	0 (217)	0 (131)	1 (244)
With IUD/IUS (n)^b^	10 (258)	12 (148)	5 (217)	5 (131)	5 (244)
With vaginal ring (n)^b^	0 (258)	8 (148)	6 (217)	2 (131)	5 (244)
With transdermal patch (n)^b^	0 (258)	3 (148)	3 (217)	2 (131)	4 (244)
**Common side effects experienced with the contraceptive pill,%**	(n=202)^c^	(n=117)^c^	(n=189)^c^	(n=114)^c^	(n=198)^c^
Weight gain	51	44	50	51	45
Mood swings	39	35	30	29	38
Headaches	25	26	32	27	26
Irritability	26	26	21	16	33
Change in libido	24	30	30	7	23
Breast tenderness/enlargement	24	26	26	16	22
Short temper	23	11	20	14	24
Migraines	22	21	17	20	14
Pre-menstrual bloating	22	9	14	15	15
Pre-menstrual syndrome	20	13	10	4	20

*Incidence of side effects with methods not listed was ≤1% in all countries.

^a^Number of women responding to the question;

^b^Total number of women who experienced side effects either with their current or previous method.

^c^Total number of women experiencing side effects, currently or previously, with the contraceptive pill.

### Women’s reasons for changing their contraceptive method

**Figure 2:**
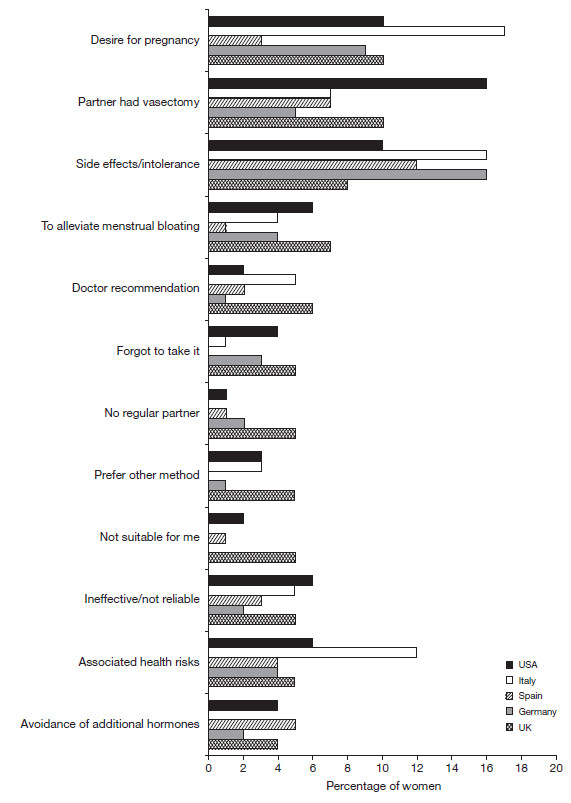
**Stated reasons why women have changed their method of contraception.** UK, n=244; Germany, n=192; Spain, n=182; Italy, n=76; USA, n=249.

Of those women who had previously used more than one method of contraception, the most common reasons for changing their method were a desire to become pregnant, intolerance of side effects, health risks associated with use and a partner having undergone a vasectomy procedure (Figure 2). Overall, side effects were the main reason women stated that they had switched from the contraceptive pill, but ranking varied among countries (most common reason in Spain [21%], Italy [26%] and the USA [18%] and the second most common in Germany [15%] and the UK [10%]).

Of women who had reported experiencing side effects with contraceptive use, approximately 80% were users (current or previous) of the contraceptive pill (Table 2), with weight gain (range 44% [Germany]–51% [Italy/UK]) and mood swings (range 29% [Italy]–39% [UK]) the most commonly experienced symptoms. Convenience was the most cited reason for switching from using male condoms in Germany (24%) and Spain (29%), and wanting more security was the most commonly stated reason in Italy (38%). Sterilisation (partner had vasectomy 29% USA, 11% UK, 9% Spain, 7% Germany, 0% Italy) or planning pregnancy (15% Italy, 14% USA, 11% UK, 5% Germany, 4% Spain) were some of the other reasons women stated that they had switched from using condoms (UK, n=56; Germany, n=41; Spain, n=68; Italy, n=13; USA, n=65).

**Figure 3:**
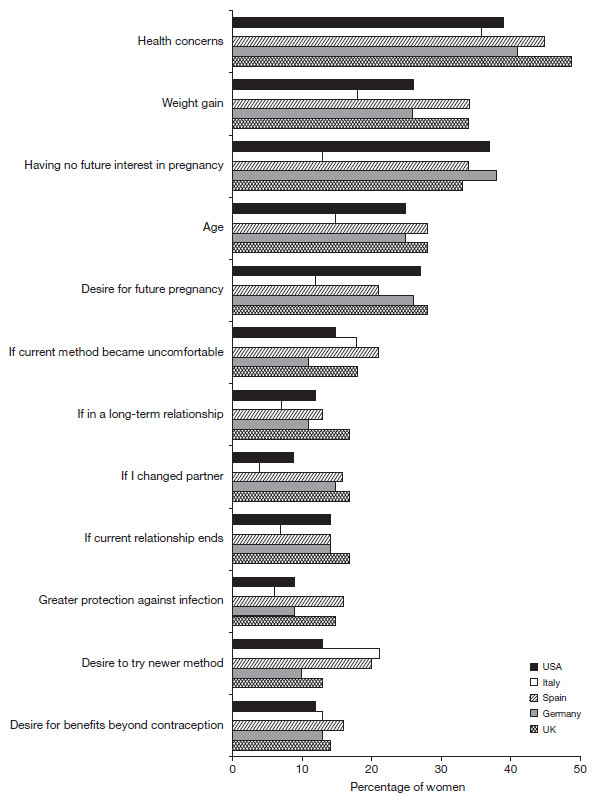
**Considerations that would prompt women to change their method of birth control.** (Q. Which of the following reasons would prompt you to consider changing your birth control method or begin using a method of birth control, if any?) UK, n=507; Germany, n=514; Spain, n=510; Italy, n=503; USA, n=507.

Sixty two percent of Spanish women reported that they were likely to change their contraceptive method within the next 0–5 years (36% of these within the next 12 months); 43% in the USA, 42% in the UK, 38% in Germany and 28% in Italy. Health concerns was the main reason women stated that they would switch methods (Figure 3), and a high percentage of women in all countries stated that they were concerned by side effects or health problems generated by methods of contraception (UK 70%, Germany 52%, Spain 96%, Italy 58%, USA 69%).

### Women’s awareness of contraceptive methods

**Figure 4:**
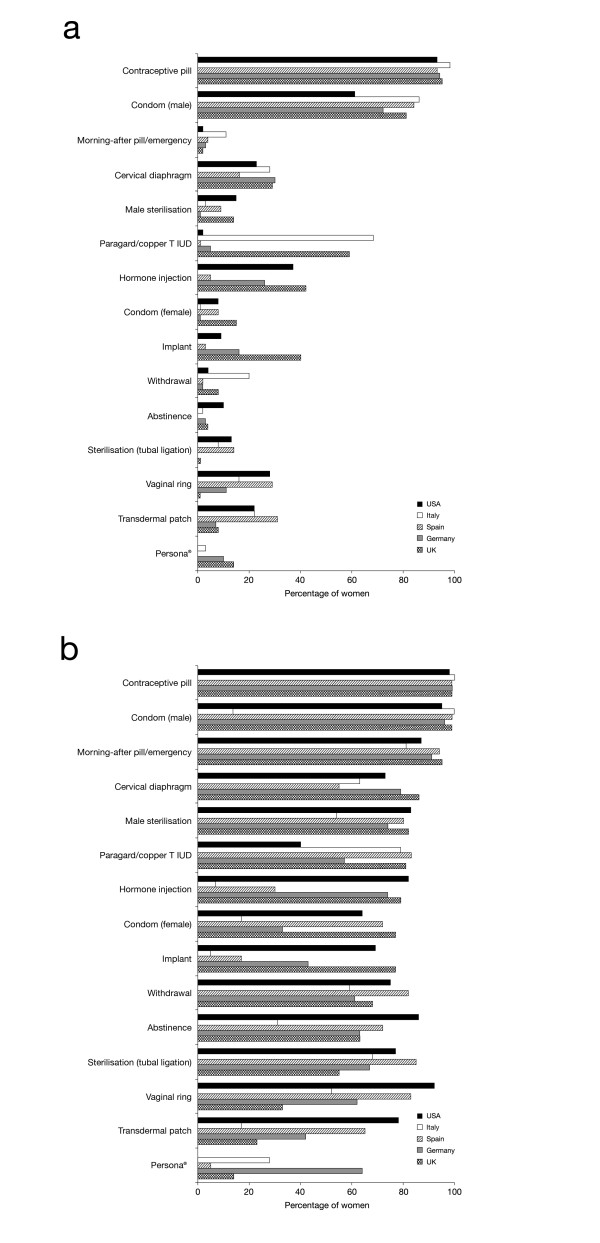
**Women’s awareness of methods of contraception. a**. Women’s unprompted awareness of contraceptive methods (Q. What are ALL the methods of birth control you have ever heard of?). **b**: Women’s prompted awareness of contraceptive methods (Q. From the list of birth control methods below, please select the ones you have EVER HEARD OF or READ about) UK, n=510; Germany, n=514; Spain, n=510; Italy, n=503; USA, n=507 (note Persona® is not available in the USA).

The contraceptive pill was the method most commonly stated as the first to come to mind when thinking about birth control by women in all countries; UK, 72%; Germany, 76%; Spain, 51%; Italy, 59%; USA, 63%. Women’s unprompted awareness of other more natural, hormone-free, methods of contraception varied; awareness of the cervical diaphragm was between 16% (Spain)–30% (Germany), awareness of the female condom was ≤15% and awareness of Persona® was ≤10% (Figure 4a). However, when asked to select which contraceptive methods they were aware of from a list, there was greater awareness of all forms of contraception (Figure 4b); for example awareness of the cervical diaphragm increased to 55% (Spain)–86% (UK).

### Sources of advice and information on contraception

Doctors, either general practitioners or specialists depending on the local health system, have the greatest influence on what type of contraception women choose (>50% of women in all countries) (Table 4). The methods of contraception most recommended to women by doctors are the contraceptive pill (range 69% in the USA/Spain to 77% in Germany) and male condoms (range 28% in Italy to 53% in Spain).

**Table 4:** Sources of contraceptive advice and information for women

	**UK**	**Germany**	**Spain**	**Italy**	**USA**
	**n=510**	**n=514**	**n=510**	**n=503**	**n=507**
**Most influence on choice of contraception,%**					
GP/primary care physician	46	1	3	5	9
Doctor/gynaecologist/obstetrician	15	65	75	72	50
Nurse (nurse practitioner, family planning nurse)	8	0	1	1	0
Pharmacists	0	0	1	0	0
Family/friends	3	4	3	2	4
Partner/spouse	17	18	14	18	24
Other	9	11	3	1	13
**Source of advice on methods of contraception,%**					
Healthcare professionals	52	57	59	71	56
Internet	32	39	26	13	26
Partner/family or friends	20	27	34	35	28
Articles/advertisements/books	9	16	23	13	14
Brochures in doctor’s waiting room	10	29	17	12	14
Family planning organisation/clinic	15	1	17	12	5

The internet and family/friends are other commonly named sources of information on contraception for women (Table 4). However, the use of the internet is lower in Italy compared with other countries and a high percentage of women in Germany stated that they obtained information from brochures in doctors’ surgeries.

## Discussion

This study found that women aged 25–44 are aware of a wide variety of contraceptive methods, but the contraceptive pill and condoms are most well known and used. It was also found that women often change their contraceptive choice, with many reasons provided, including planning pregnancy, and for pill users, concerns about side effects. Healthcare professionals were the main source of advice. There were country-specific differences in women’s choice and awareness.

The contraceptive pill and male condom (particularly in Southern Europe) are the main methods of contraception used across the five participating countries; this is in agreement with previously published studies [1,2,5,6]. These two methods are also the most well-known and recommended by healthcare professionals. Although contraceptive choice has increased in recent years, women’s unaided awareness of newer methods such as the vaginal ring is low (<30%), partly due to the fact that not all new methods are readily available in all countries or regions and because they are not typically recommended by healthcare professionals. However, when provided with a list of available forms of contraception, respondents recognised most methods.

Satisfaction with the current method of contraception is high across countries, in agreement with another recent study conducted in the UK [7], yet 28–62% of women expected to switch methods in the next 5 years, with health concerns being one of the main reasons. The concern about side effects is relatively high across all countries, and women are most concerned about hormonal methods. Currently 5–20% of women report that they have experienced side effects with their chosen method of contraception.

It is interesting to note that mood swings and weight gain are commonly stated as side effects experienced when using the contraceptive pill, yet clinical studies have failed to confirm these associations. Studies have shown no link exists between the contraceptive pill and mood swings and, in fact, there is evidence to suggest that the contraceptive pill may even improve or stabilise moods [8-15]; despite this, a recent survey of UK healthcare professionals found that 87% believed that the contraceptive pill could cause mood changes [16]. In addition, studies on weight gain have found that women’s weight remains essentially unchanged while taking the contraceptive pill, with only minor increases or decreases observed [17]. This indicates that many myths about contraceptive methods persist and that women, and possibly healthcare providers, are not properly informed about contraceptive methods.

Although women state that their main reason for changing their method of contraception is health concerns, especially with the contraceptive pill, women’s awareness of non-hormonal methods of contraception other than male condoms is low; ≤15% of women were aware of methods such as the female condom or Persona®. This finding suggests that information on alternative methods of contraception is not readily available to women and/or they do not actively seek advice on alternative methods. Limitations of this study include the low sample size when examining responses from users of less common types of contraception.

Women reported that their doctor (either general practitioner or specialist) is the major influence on their choice of contraception; with the exception of Spain, pharmacists are rarely consulted. The CHOICE study (Contraceptive Health Research of Informed Choice Experience) reported that structured and balanced counselling of women aged 15–40 years, who consulted their healthcare professional about contraception, led to more than 40% of women changing their mind on the mode of delivery of hormonal contraception from their initial choice [18]. Women’s contraception needs change during the course of their life [6]; it is therefore important that they have access to all the necessary information to help them make the right choice at the appropriate stage in their life. Healthcare professionals need to inform their patients about the benefits and risks of all available contraceptives. This may prompt women to reconsider their original choice, potentially selecting a method which better suits their medical and lifestyle needs. Increasing women’s awareness of alternative hormone-free methods of contraception would assist women to make a more informed choice, particularly when considering a change due to concerns over side effects associated with hormonal methods.

## Conclusions

Women aged 25–44 are aware of a wide variety of contraceptive methods, but knowledge and usage of the contraceptive pill and male condom predominates. Changing contraception method is frequent, occurring for a variety of reasons, including change in life circumstances and, for pill users, concerns about side effects.

## Competing interests

The study was funded by SPD Swiss Precision Diagnostics GmbH, of which Christine Pion is an employee. Sarah Johnson is an employee of SPD Development Company Ltd. (a wholly owned subsidiary of SPD Swiss Precision Diagnostics GmbH). Victoria Jennings is an independent researcher, who did not receive honoraria for her contribution to this study, but has provided consultancy to SPD Swiss Precision Diagnostics GmbH.

## Authors’ contributions

SJ, CP and VJ were involved in the design of the study together with data analysis/interpretation and preparation of the manuscript. All authors read and approved the final manuscript.
